# Influence of government policies on handwashing and vaccine uptake in Kenya, Uganda, and Tanzania to prevent and control COVID-19: a systematic review

**DOI:** 10.3389/fpubh.2024.1417866

**Published:** 2024-12-18

**Authors:** Josphat Martin Muchangi, James Mturi, Hajra Mukasa, Kioko Kithuki, Sarah Jebet Kosgei, Lennah Muhoja Kanyangi, Rogers Moraro, Samuel Kamau, Maureen Nankanja

**Affiliations:** ^1^Amref Health Africa, Nairobi, Kenya; ^2^Amref Health Africa, Dar es Salaam, Tanzania; ^3^Amref Health Africa, Kampala, Uganda; ^4^Reinit Research, Nairobi, Kenya

**Keywords:** handwashing, COVID-19 vaccine, uptake, policy, government, Kenya, Uganda, Tanzania

## Abstract

**Introduction:**

The government's role in influencing policies related to Coronavirus disease 2019 (COVID-19) vaccine distribution and handwashing practices is essential in controlling the spread of severe acute respiratory syndrome coronavirus 2.

**Methods:**

This study aimed to systematically review published studies to explore the influence of government policies on handwashing and vaccine uptake in Kenya, Uganda and Tanzania to prevent and control COVID-19. A comprehensive search strategy was applied across three databases, and eligibility was determined using strict inclusion and exclusion criteria. We reviewed 9 of 136 research papers following the Preferred Reporting Items for Systematic Review and Meta-Analysis (PRISMA) guidelines.

**Results:**

The findings revealed that the government has a role in influencing policies related to COVID-19 vaccine distribution and handwashing uptake. Employment of public health campaigns and communication strategies by the government in Uganda increased vaccine acceptance and hand hygiene uptake. Similarly, government efforts to make hand hygiene accessible increased the uptake of handwashing in Tanzania. In Kenya, government efforts to increase access to soap and clean water in informal settlements and markets resulted in increased adherence to handwashing practices. Further, government incentives such as cash increased vaccination rates while vaccination reminders combined with cash incentives increased childhood immunization coverage.

**Discussion:**

Overall, this review indicates that monitoring and enforcing compliance increases vaccine and handwashing uptake across the three countries. The effectiveness of government policies on handwashing and vaccine uptake is influenced by factors such as safety, efficacy and access to information, among others. Therefore, there is a need to address these factors for the successful implementation of these policies.

**Systematic review Registration:**

PROSPERO ID CRD42023396319, https://www.crd.york.ac.uk/prospero/.

## Background

The coronavirus disease 2019 (COVID-19) pandemic has led to loss of lives and severely crimpled economies globally (1). At the same time, people have raised social issues including the erosion of public trust in government efforts and expert advice due to misrepresentation and misinformation (2, 3). Whilst the development of COVID-19 vaccines is an extraordinary achievement, successful vaccination of populations and adherence to proper handwashing practices face challenges associated with production, distribution, and acceptance (4). The government's role in influencing policies related to COVID-19 vaccine distribution and handwashing uptake is essential in controlling the spread of the virus and protecting public health (5).

The effective implementation of COVID-19 policies requires public compliance. Of note, public compliance depends on the trust people have in government actions, hence its importance in the success of any government project (6, 7). Government actions include public scrutiny and public participation, equitable distribution of COVID-19 vaccines and handwashing stations (8). Trust in government institutions involved in the distribution of vaccines, monitoring and information reflects people's satisfaction with the government's policy and performance (6)^1^.

The current infrastructure and supplies are inadequate to warrant a swift vaccination campaign when it comes to the transport and storage of vaccines in most places (9). Several governments have made efforts to ensure the timely delivery of COVID-19 supplies by developing policies and infrastructures for distributing, storing and administering vaccines across their jurisdiction (9). In the United States of America, each state has devised a vaccine tracking system where each state orders doses up to a limit decided at the federal level, making vaccines and other COVID-19 supplies accessible (10–12).

Collaboration between the public and private sectors as well as other international bodies is key in ensuring that the COVID-19 supplies and services are supplied based on risk, resource, and benefit sharing (13, 14). In Zambia, the government has leveraged local and international support to combat the COVID-19 pandemic. The collaboration was seen in the sensitization of Zambian citizens about the pandemic through messages and advertisements distributed through radio, television, and telephone companies (15). In addition, collaboration between academia, government, and the community is crucial in promoting health fairness (16). Further, the government and community collaboration ensures more community members receive vaccinations, raising the total immunization rate.

Effective communication by the government is key for handwashing and COVID-19 uptake to succeed, hence minimizing hesitancy (15). A study to establish why Egyptian medical students were reluctant to receive vaccinations reported that the government should create more awareness about the importance of vaccination to increase vaccination adoption (17). To comprehend vaccine reluctance, investigation of vaccine roll-out in South Africa and Zimbabwe emphasized the importance of the government in disseminating information and knowledge about vaccines to stop false information from stifling vaccination rates (18). Assessment of vaccine reluctance in South Africa from 2020 to 2021 revealed that communication campaigns and other forms of community engagement are key in addressing some of the concerns, hence building people's trust in handwashing practices and vaccine acceptance (19).

Government incentives such as cash rewards, free meals, or reduced transportation costs can encourage people to get vaccinated. An increase in government spending in a program to increase the number of free vaccines available to citizens who willingly underwent mass vaccination has been previously shown to enhance vaccine uptake in nations with low and intermediate incomes (20). Local and federal governments in the United States of America offered financial incentives, including gift cards, a lottery with a million-dollar top prize, and savings bonds to entice people to get immunized (21). Another study indicated that financial incentives had raised vaccination rates among Swedish people (22). Government policies play a key role in shaping vaccine policy and availability, as well as in implementing programs and influencing vaccine acceptance in society.

However, literature supporting this notion remains scarce in sub-Saharan Africa, where the uptake of COVID-19 vaccine and handwashing remains low. For instance, in Tanzania, a community based survey found only 18% of respondents had received COVID-19 vaccine (38) while a longitudinal study across 10 sub-Saharan African countries observed a significant decline in handwashing prevalence between July and November 2020 (39). Therefore, this study aims to investigate the role of government policies in promoting key public health behaviors, specifically the uptake of COVID-19 vaccines and handwashing practices, as part of the broader effort to control the spread of COVID-19 in sub-Saharan Africa. The focus of this research is on how governments can enhance prevention through public health campaigns, ensure compliance with health guidelines, and guarantee the availability of essential materials and tools such as soap and vaccine doses. By analyzing the impact of these policies, the study seeks to identify effective strategies for increasing both vaccination rates and adherence to hand hygiene practices, which are critical in preventing the transmission of COVID-19.

## Materials and methods

### Study design

This review was undertaken in accordance with the Preferred Reporting Items for Systematic Reviews and Meta-Analyses (PRISMA) and the Centre for Reviews and Dissemination (CRD) guidelines (23, 24). The protocol was registered at the International Prospective Register of Systematic Reviews (PROSPERO) database with approval ID CRD42023396319.

### Search strategies

A systematic search of the literature was conducted using PubMed and ScienceDirect as the primary databases. We also used Google Scholar as a supplementary tool to identify additional relevant studies that the primary databases may not have captured. A search strategy was formulated based on the PECOS framework by combining all possible combinations (Supplementary Tables 1, 2). The following search terms and their synonyms using Boolean operators were used to perform the search strategy: handwashing, vaccine, COVID-19, prevention, policy, Kenya, Uganda, and Tanzania.

### Inclusion criteria

Our review considered all published studies focusing on the influence of government policies on COVID-19 vaccination and handwashing conducted in Kenya, Uganda, and Tanzania. There was no restriction on the publication date or language.

### Exclusion criteria

We excluded studies that did not address the influence of government policies on handwashing and vaccine uptake in Kenya, Uganda, and Tanzania to prevent and control COVID-19. In addition, literature reviews and preprints were ineligible for inclusion in this systematic review.

### Data screening and selection

We selected studies in two stages after the initial removal of duplicates. First, titles and abstracts of the retrieved articles were screened for relevance by two independent reviewers, JM and KK. The full texts of potentially relevant studies were further assessed for data extraction. We used Mendeley for reference management of the potentially relevant articles. Disagreements were resolved through discussion with a third reviewer, RM. We ensured internal consistency by conducting training for reviewers to ensure they understand the criteria and process.

### Quality assessment and risk of bias

JM and KK assessed the potential for bias in the eligible articles based on the Quality Assessment Tool for Observational Cohort and Cross-sectional studies (https://www.nhlbi.nih.gov/health-topics/study-quality-assessment-tools) (25). This checklist encompasses 14 crucial criteria regarded as fundamental for ensuring the quality of reporting in cohort and cross-sectional studies. These recommendations focus on various aspects, including the article's objectives, the study population, exposure measures and potential confounders, among others.

### Data extraction

JM and KK extracted data from the selected studies using a standardized data extraction form, and any disagreements were resolved by consensus. The data extracted included the name of the first author(s) and year of publication, study title, country of study, study objective(s), study design, outcome definition, and main findings on how government policies have influenced COVID-19 vaccine and handwashing uptake as an intervention.

### Data synthesis

We used narrative synthesis to summarize the main results of the eligible studies. A table was used to show the study characteristics and indicate how government policies influence handwashing and vaccine uptake in Kenya, Uganda, and Tanzania to prevent and control COVID-19.

## Results

### Study selection

**Figure 1:**
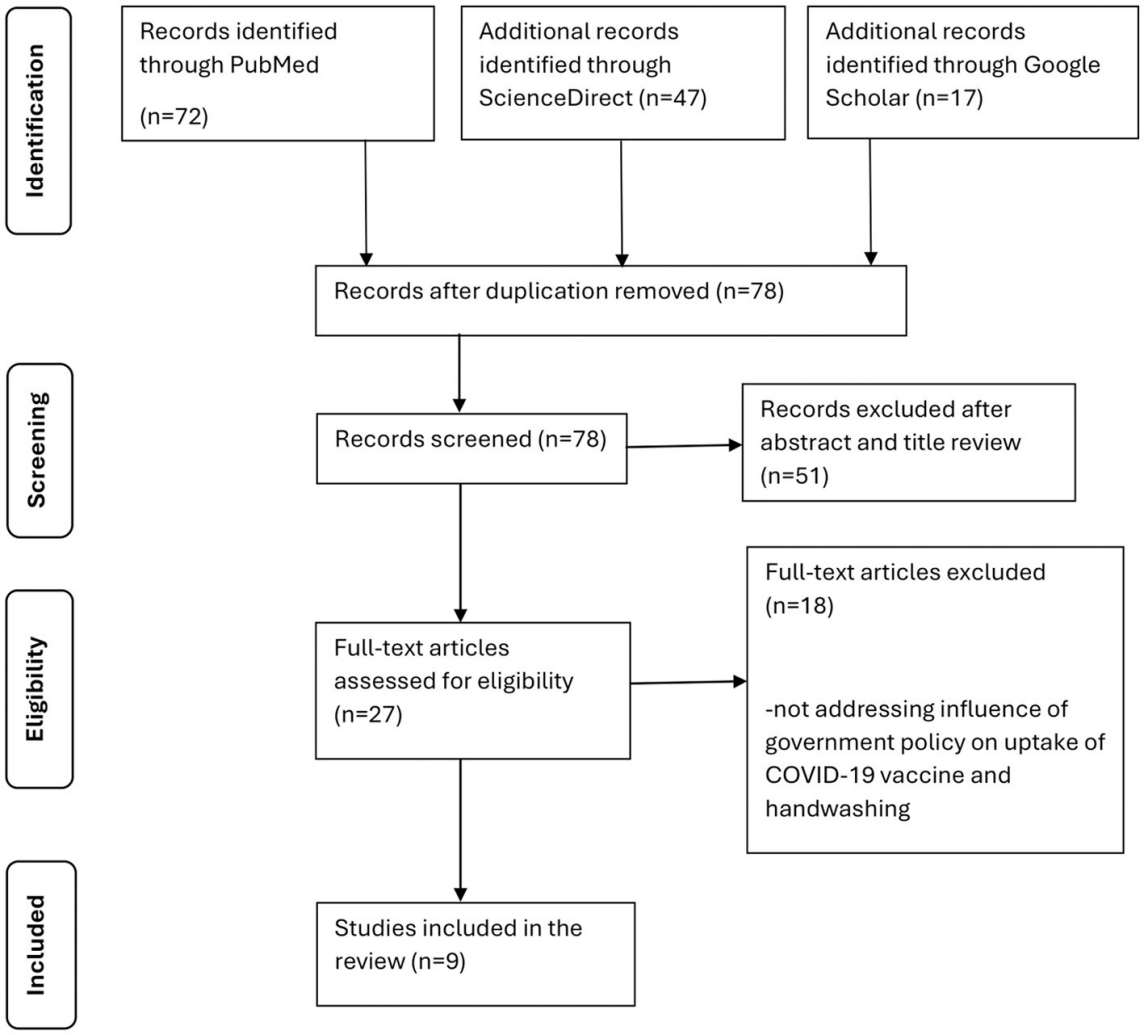
PRISMA chart depicting the study selection process.

We identified 136 research papers from our database searches, as summarized by Figure 1. After removing duplicates, 78 papers underwent abstract and title screening, which resulted in the exclusion of 51 studies. The remaining 27 studies underwent full-text screening, 18 of which were excluded due to irrelevant study outcomes. This systematic review includes 9 articles focusing on the influence of government policies on vaccine uptake and handwashing to prevent and control COVID-19.

### Characteristics of the studies

The main characteristics of the studies included in this systematic review are presented in Table 1. Three of the eligible studies focused on Kenya, with Tanzania and Uganda having two and four studies, respectively. Publication years ranged from 2021 to 2022. The study designs consisted of cross-sectional studies, multi-sited historically informed ethnographic fieldwork, and qualitative interviews. The study participants ranged from pregnant and lactating women to health workers, policymakers, and institutions such as supermarkets. Similarly, the eligible studies' sample sizes ranged from 33 to 2,245.

**Table 1:** The characteristics of the eligible studies.

**References**	**Title of paper**	**Country**	**Study design**	**Sample size**	**Participants**	**Age [mean (SD)]**	**Dates of data collection**	**Outcome**	**Outcome definition**	**Main findings**	**Risk of bias**
Blair et al. (26)	Public trust, policing, and the COVID-19 pandemic: Evidence from an electoral authoritarian regime	Uganda	Mobile phone panel survey	2245	Respondents across 288 villages spanning 13 districts and all four regions of Uganda	Not available	July and September 2020	Vaccine acceptance	The level of influence by leaders at all levels to help debunk fears and misconceptions about the AstraZeneca vaccine and to encourage their followers to get vaccinated	Messages from government officials generated a stronger support for public health restrictions.	Low^*^
Chilongola et al. (27)	COVID-19 Knowledge, Attitudes, Practices, and vaccination hesitancy in Moshi, Kilimanjaro Region, Northern Tanzania	Tanzania	Cross-sectional study	232	Individuals who visited their relatives who were admitted or undergoing medical care at Kilimanjaro Christian Medical Centre were requested to respond to structured questions regarding COVID-19.	33 (25–45) years	October 2021	The proportion of participants who were unwilling to take COVID-19 vaccine	152 (65.52%) of interviewed participants had a negative attitude toward COVID-19 vaccines.	5.2% of the participants indicated that the first-time hearing about COVID-19 was from the government ministry	Low^*^
Harris et al. (32)	Improving the effectiveness of Field Epidemiology Training Programs: characteristics that facilitated effective response to the COVID-19 pandemic in Uganda	Uganda	Descriptive study	188	Field Epidemiology Training Programs	Not applicable	February 2020–September 2021	COVID-19 pandemic response	COVID-19 response activities requested by the ministry of health and other stakeholders	Some of the activities included rollout and management of contact tracing and data management, healthcare worker infection surveillance and risk mapping	Low^*^
Konje et al. (28)	The Coverage and Acceptance Spectrum of COVID-19 Vaccines among Healthcare Professionals in Western Tanzania: What Can We Learn from This Pandemic?	Tanzania	Cross-sectional study	811	Health professionals	35 (9) years	September 2021	Factors associated with COVID-19 vaccine uptake	Cues for actions on improving COVID-19 vaccine uptake among health professionals	A majority of participants reported that engagement of government authority for the provision of vaccine information, involvement of public figures in advocacy of the vaccine, and support from close family members and friends would improve the vaccine's uptake.	Low^*^
Mugambe et al. (34)	Extent of compliance with COVID-19 prevention and control guidelines among supermarkets in Kampala Capital City and Mukono Municipality, Uganda	Uganda	Cross-sectional study	229 supermarkets	Supermarkets in Kampala Capital City and Mukono Municipality	Not applicable	September 2020	Compliance with the COVID-19 prevention guidelines on handwashing	Extent of compliance with the COVID-19 prevention guidelines on uptake of handwashing	Only 16.6% (38/229) of the supermarkets complied with the COVID-19 prevention and control guidelines. Only 40.6% and 30.6% of the supermarkets enforced mandatory handwashing and use of face masks respectively for all customers accessing the premises.	Low^*^
Okedi et al. (33)	The Impact and Sustainability of Hand-hygiene Practices on Health Facilities in the Context of the COVID-19 Pandemic. A Case Study of Busia County (Kenya)	Kenya	Cross-sectional study	33 health facilities	Medical officers, nursing officers, public health officers, registered clinical officers, laboratory technologists, pharmaceutical technologists, nurse attendants and subordinate staff of health facilities	Not available	Not available	Hand hygiene practices	Compliance with hand hygiene guidelines	Only 2 (28.6%) of facilities enforced obligatory use of hand hygiene and there was no policy on hand hygiene in 6 (86%) health facilities.	Low^*^
Orangi et al. (30)	Epidemiological impact and costeffectiveness analysis of COVID-19 vaccination in Kenya	Kenya	Cost-effectiveness analysis	Not applicable	Not applicable	Not applicable	September 2021–February 2023	Vaccine uptake	Epidemiological impact and cost effectiveness of a range of COVID-19 vaccine deployment strategies and scenarios in Kenya	A strategy to vaccinate mostly older adults (80% of those over 50 years) who are at high risk of severe disease but which achieves low (30%) overall population coverage, yields the greatest reductions in severe infections and deaths per fully vaccinated adult.	Low^*^
Parker et al. (31)	Epidemics and the Military: Responding to COVID-19 in Uganda	Uganda	Multi-sited, historically informed ethnographic fieldwork				July and September 2020	Vaccine uptake	The extent to which military can influence vaccine uptake	Vaccines were used on traders at regulated crossing points each time they crossed, if proof of vaccination could not be produced.	Moderate^**^
Zavala et al. (29)	Lack of clear national policy guidance on COVID-19 vaccines influences behaviors in pregnant and lactating women in Kenya	Kenya	Qualitative interviews	59	Pregnant and lactating women, health workers and policy markers	Not available	August 8th–September 3rd, 2021	Perceptions of national policy regarding COVID-19 vaccination in pregnancy	Perceptions of national policy regarding COVID-19 vaccination in pregnancy and how they shape vaccine behaviors and decision-making	Policymakers and health workers described pervasive uncertainty and lack of communication about the national policy, cited vaccine safety as their primary concern for administering COVID19 vaccines to PLW, and expressed that PLW were inadequately prioritized in the COVID-19 vaccine program.	Moderate^**^

* All quality criteria were fulfilled, indicating a low risk. ^**^Lack of sample size justification is a limitation that raises moderate risk.

### Quality evaluation

According to the Quality Assessment Tool for Observational Cohort and Cross-sectional Studies checklist, all the studies met the recommendations for conducting observational studies (Supplementary Table 3). This finding indicates high overall methodological quality and low risk of bias in these studies.

### Influence of government policies on handwashing and vaccine uptake

The following results are organized into key themes that emerged during data extraction and synthesis. These themes represent the various ways in which government policies have influenced COVID-19 vaccine uptake and handwashing practices. The themes include public health campaigns and communication strategies, accessibility of vaccines and hand hygiene supplies, vaccine certification, collaborations with community leaders, and monitoring and enforcement of compliance. These categories emerged organically from the analysis of the studies, allowing us to systematically assess the diverse impact of government interventions on these preventive behaviors.

#### Public health campaigns and communication strategies

Four studies in the systematic literature review have revealed that the government's efforts to promote public health campaigns and communication strategies to educate the public about COVID-19 have led to vaccine acceptance and handwashing uptake. A Ugandan study reported that messages from government officials generated a stronger support for public health restrictions (26). Governments can influence the control and prevention of COVID-19 by making citizens aware of the disease. Chilongola et al. reported that 5.2% of the participants first heard about COVID-19 from the government ministry after assessing COVID-19 knowledge (27). The engagement of government authorities in the provision of vaccine information was also reported as a potential factor in improving COVID-19 vaccine uptake (28). Conversely, a study involving pregnant and lactating women (PLW) showed uncertainty and lack of communication about the national policy, with healthcare workers citing vaccine safety as their primary concern for administering COVID-19 vaccines to this population (29). As such, there was a perception by the PLW of the restrictive policy as an indicator of a safety risk, resulting in vaccine hesitancy and potentially exacerbated inequities in vaccine access (29).

#### Making COVID-19 vaccines and hand hygiene accessible was found to influence uptake of vaccines

Increased accessibility of vaccines and hand hygiene supplies by the government can increase uptake of COVID-19 vaccine and handwashing practices. For instance, a cost-effectiveness analysis conducted in Kenya showed that vaccine deployment strategies targeting those at risk of disease and other vulnerable groups rather than the whole population could increase vaccine uptake (30). One important strategy is to ensure that there is an appropriate supply of the vaccine in all locations and that it is distributed fairly to all populations, regardless of their socioeconomic situation or geography.

#### Vaccine certification/verifying vaccination status through proof-of-vaccine

A study to examine the extent to which the military can influence vaccine uptake in Uganda revealed soldiers stationed on the ground exerted authority by making people without vaccine cards be vaccinated (31). As such, government policies requiring vaccination for specific activities; and verifying vaccination status through proof-of-vaccine programs can increase vaccination rates. Putting such a policy into practice at the local or national level and in particular professions, like healthcare, education, or travel, would be crucial in raising vaccination rates.

#### Partnering with community leaders and organizations

Harris et al. highlighted government policies that allow collaborations with community leaders in awareness creation about COVID-19 could build trust. Strong leadership and collaborations, as witnessed in Uganda, are critical in building trust and influencing handwashing and COVID-19 vaccine uptake (32).

#### Monitoring and enforcing compliance of COVID-19 related measures and guidelines

Two studies involving 33 health facilities and 229 supermarkets indicated that monitoring and enforcement of vaccine-related policies is key in raising the uptake of handwashing and vaccines (33, 34). The findings revealed that only one hospital had a national policy on hand hygiene. The majority of the respondents indicated that enabling factors for implementing hand hygiene include a clear commitment of national and county governments to providing clear policy frameworks and guidelines that support hand hygiene initiatives (33). Similarly, an assessment of compliance levels among supermarkets in Kampala and Mukono Municipality indicated that the supermarkets complied with the COVID-19 prevention and control guidelines, including the establishment of handwashing stations, with more than half of the supermarkets having someone or a team in charge of enforcing compliance to COVID-19 measures (34).

## Discussion

Our study shows that the government has an essential role in influencing policies related to COVID-19 vaccine distribution and handwashing uptake to control its spread. The government's employment of public health campaigns and communication strategies to educate the public about the safety of COVID-19 vaccines and hand hygiene practices has led to increased uptake. These findings are consistent with previous research where awareness creation by the government about vaccines was shown to improve vaccine acceptance (17). Similar findings emphasized the government's role in creating awareness to reduce vaccine hesitancy (18, 19). Campaigns and community engagement activities can increase people's confidence in the safety and efficacy of COVID-19 vaccines by addressing the associated concerns and the importance of hand hygiene.

Government involvement in increasing vaccine availability and hand hygiene supplies can increase the COVID-19 vaccine and handwashing uptake. The results are consistent with the literature, which notes that vaccine uptake could be improved by increasing government budgets (20). Additionally, this finding is parallel with global experiences beyond the COVID-19 pandemic. For instance, in Australia, the introduction of funded influenza vaccine programs for children under 5 years old led to a significant increase in vaccine uptake, with a 2.7- to 4.2-fold rise observed. Importantly, this initiative also had spillover effects, enhancing vaccine coverage among older children and adults (40). Such initiatives are aimed at increasing the availability of free vaccines among citizens who voluntarily undergo vaccination in large numbers. As such, policymakers can invest in minimizing the scarcity of vaccines, boosting vaccine intention and vaccination rates among populations.

A critical factor in understanding the outcomes of government interventions is the level of exposure to these policies and the resources available to support them. In countries with higher levels of public health funding and infrastructure, strict containment measures accompanied by effective communication and resource distribution have led to improved handwashing adherence and vaccine uptake. For instance, studies from high-income countries like Australia and Sweden demonstrate that well-resourced government programs yield positive outcomes in both hand hygiene and vaccination rates (22, 40).

However, in low- and middle-income countries (LMICs), where strict policies were not always accompanied by adequate resources, adherence to these behaviors was lower, often due to limited access to necessary supplies or a lack of public trust (42). In some cases, stricter containment measures, without sufficient support, or communication, resulted in public frustration and lower adherence.

Moreover, findings from a study conducted in 14 countries suggested that strict government policies during the COVID-19 pandemic may have inadvertently undermined individual self-regulatory processes, reducing handwashing adherence. More lenient policies, on the other hand, may have prompted greater self-regulatory efforts, as individuals faced higher-risk situations and were motivated to adopt protective behaviors (44). Additionally, government incentives such as cash rewards can encourage people to get vaccinated, thus increasing vaccination rates. This outcome is in concordance with previous research which highlighted that financial incentives and other nudges amplified vaccination rates in the United States of America (21). In a different setting, an assessment of monetary incentives in relation to vaccination rates among Swedish individuals revealed that the use of incentives increased vaccination rates by about four points (22). Monetary compensation to individuals who accept vaccination and adhere to proper handwashing practices can help in controlling the spread of COVID-19.

Previous research has also explored how proof of vaccination in accessing government services increases vaccination concurs with the current research findings (35). The study findings revealed that school-entry mandates increased COVID-19 vaccination rates among children. Other studies have shown that the announcement of vaccination mandates can increase vaccination rates by over 60% (36).

Government policies that allow partnering with community leaders and organizations can build trust and engage with hard-to-reach communities, thus increasing vaccination rates. For instance, collaborations between governments and the community can be used to address vaccine hesitancy (37). Other studies have revealed that faith-based wellness programs rooted in African American communities are trusted to provide accurate information. Therefore, the academic-government-community collaboration is essential in ensuring health equity (16).

Recent studies have provided valuable insights into the effectiveness of government interventions in promoting these crucial public health measures. For instance, Matkovic et al. demonstrated that simple, brief, and easily conveyable messages could positively impact behavioral intentions around handwashing during the early stages of a health crisis across the United States during the COVID-19 pandemic (41). Their findings emphasize the importance of clear, consistent messaging from authorities in promoting proper hand hygiene. Additionally, a systematic review of handwashing interventions in low- and middle-income countries highlighted the importance of multi-level interventions and the combination of training, policy, and funding strategies in implementing effective hand hygiene programs (42). Regarding vaccine uptake, studies have examined the impact of various government policies, revealing that state-level mandates and incentives significantly increase vaccination rates (36, 43). This aligns with our findings on the effectiveness of government incentives and mandates in boosting vaccine acceptance.

The study's findings also imply that the government seeking to increase vaccination rates must collaborate with community-based organizations to increase awareness of vaccines and vaccination centers.

### Strengths and limitations

This systematic review has some strengths: first, we used a comprehensive search strategy to identify eligible research papers, and our protocol was registered on the PROSPERO database. We did not limit our search based on publication date or language of publication, thereby increasing the number of studies identified. Despite these advantages, it's important to acknowledge our study's limitations. The heterogeneity of the included studies and potential publication bias may affect the generalizability of our findings. We employed a rigorous methodology to mitigate these issues, including a comprehensive search strategy and independent screening by multiple reviewers. However, future research should consider conducting primary studies with standardized measures to further validate the effectiveness of government policies on handwashing and vaccine uptake. Moreover, we only focused on Kenya, Uganda, and Tanzania; hence, the results may not be generalizable to other areas due to differing political, environmental, economic, and social characteristics. Additionally, we recognize the need to explore the long-term sustainability of government interventions and their ethical implications, particularly concerning mandates and incentives. These aspects call for further investigation to ensure the development of effective and ethically sound public health policies.

## Conclusions

In conclusion, government policies that promote access to accurate information about hand hygiene through health campaigns and the provision of water and soap have been shown to be effective in increasing handwashing practices among the general public. The requirement to verify vaccination status, provision of cash incentives, and government partnership with community leaders and organizations help build trust and improve vaccine uptake rates. Therefore, policymakers should not only formulate but also implement government policies that employ the identified strategies to significantly enhance the uptake of preventive measures and contribute to controlling and preventing COVID-19 in Kenya, Uganda, Tanzania, and similar regions. While the COVID-19 pandemic is no longer classified as an emergency, these strategies could be crucial in the event of a resurgence of COVID-19 or the emergence of a new virus with similar transmission characteristics. Policymakers can apply these lessons to future public health challenges to improve preparedness and response to pandemics.

## Data Availability

The original contributions presented in the study are included in the article/Supplementary material, further inquiries can be directed to the corresponding author.

## Footnotes

^1^ https://www.cdc.gov/coronavirus/2019-ncov/prevent-getting-sick/prevention.html
