# Supplementary material for: Influence of government policies on handwashing and vaccine uptake in Kenya, Uganda, and Tanzania to prevent and control COVID-19: a systematic review
Source: Front Public Health. 2024 Dec 18;12:1417866. doi: 10.3389/fpubh.2024.1417866 (PMC11689279; doi:10.3389/fpubh.2024.1417866)
Supplement: Supplementary file 1 [file Table_1.docx]

| **Supplementary table 1. Search terms and their keywords used to formulate medical subject heading (MeSH) terms.** | | |
| --- | --- | --- |
| **Term** | **Key words** | **MeSH terms** |
| Handwashing | Handwashing  Hand hygiene | ("hand disinfection"[MeSH Terms] OR Handwashing[Text Word] OR "hand hygiene"[MeSH Terms] OR hand hygiene[Text Word]) |
| Vaccination | Vaccine  Vaccination  Immunization | ("vaccination"[MeSH Terms] OR vaccination[Text Word] OR "immunization"[MeSH Terms] OR immunization[Text Word]) |
| COVID-19 | COVID-19  SARS-CoV2 | ("COVID-19"[All Fields] OR "COVID-19"[MeSH Terms] OR "SARS-CoV-2"[All Fields] OR "sars-cov-2"[MeSH Terms] OR "Severe Acute Respiratory Syndrome Coronavirus 2"[All Fields]) |
| Prevention | Prevention  Control | ("prevention and control"[Subheading] OR prevention[Text Word] OR "control groups"[MeSH Terms] OR control[Text Word]) |
| Enablers | Enablers | Enabler[Text Word] |
| Barriers | Barriers | Barrier[Text Word] |
| Policy | Policy | ("policy"[MeSH Terms] OR policy[Text Word]) |
| Government | Government | ("government"[MeSH Terms] OR government[Text Word]) |
| Kenya | Kenya | ("Kenya"[MeSH Terms] OR Kenya[Text Word]) |
| Uganda | Uganda | ("Uganda"[MeSH Terms] OR Uganda[Text Word]) |
| Tanzania | Tanzania | ("Tanzania"[MeSH Terms] OR Tanzania[Text Word]) |
| **Abbreviations and acronyms:** COVID-19, Coronavirus disease 2019; SARS-CoV2, severe acute respiratory syndrome coronavirus 2; MeSH, Medical subject heading.  We derived the keywords from the research question and used them to identify MeSH terms on PubMed database. | | |
