# Supplementary material for: Influence of government policies on handwashing and vaccine uptake in Kenya, Uganda, and Tanzania to prevent and control COVID-19: a systematic review
Source: Front Public Health. 2024 Dec 18;12:1417866. doi: 10.3389/fpubh.2024.1417866 (PMC11689279; doi:10.3389/fpubh.2024.1417866)
Supplement: Supplementary file 2 [file Table_2.docx]

| **Supplementary table 2. Search strategy used to identify eligible studies** | | |
| --- | --- | --- |
| **Database** |  | **Search strategy** |
| **PubMed** | (((term 1) OR (term 2)) AND ((term 3) AND (term 4)) AND ((term 5) OR (term 6)) AND (term 7) AND (term 8) AND ((term 9) OR (term 10) OR (term 11))) | ((("hand disinfection"[MeSH Terms] OR Handwashing[Text Word] OR "hand hygiene"[MeSH Terms] OR hand hygiene[Text Word]) OR ("vaccination"[MeSH Terms] OR vaccination[Text Word] OR "immunization"[MeSH Terms] OR immunization[Text Word])) AND (("COVID-19"[All Fields] OR "COVID-19"[MeSH Terms] OR "SARS-CoV-2"[All Fields] OR "sars-cov-2"[MeSH Terms] OR "Severe Acute Respiratory Syndrome Coronavirus 2"[All Fields]) AND ("prevention and control"[Subheading] OR prevention[Text Word] OR "control groups"[MeSH Terms] OR control[Text Word])) AND ((Enabler[Text Word]) OR (Barrier[Text Word])) AND ("policy"[MeSH Terms] OR policy[Text Word]) AND ("government"[MeSH Terms] OR government[Text Word]) AND (("Kenya"[MeSH Terms] OR Kenya[Text Word]) OR ("Uganda"[MeSH Terms] OR Uganda[Text Word]) OR ("Tanzania"[MeSH Terms] OR Tanzania[Text Word]))) |
